# Perioperative outcomes of utilizing infrahepatic inferior vena cava clamping and Pringle maneuver during hepatectomy: a meta-analysis

**DOI:** 10.1007/s00423-024-03344-6

**Published:** 2024-05-17

**Authors:** Agastya Patel, Jacob Tan, Joel Lambert, Samuel Kitching, Affan Iqbal, Thomas Satyadas

**Affiliations:** 1 Regional Hepato-Pancreato-Biliary Surgical Unit, Manchester Royal Infirmary, M13 9WL, Manchester, UK; 2 Department of General, Endocrine and Transplant Surgery, Medical University of Gdansk, Gdansk, Poland

**Keywords:** Inferior vena cava, Pringle maneuverer, Hepatectomy, Central venous pressure, Intraoperative blood loss

## Abstract

**Purpose:**

Intraoperative bleeding during hepatectomy is primarily controlled through anaesthesiological interventions or surgical techniques such as Pringle maneuver (PM). Infrahepatic IVC clamping (IIVCC) is an alternative surgical technique to reduce central venous pressure and prevent retrograde hepatic venous bleeding. The aim of the meta-analysis was to compare IIVCC+PM with PM alone in terms of intraoperative outcomes and perioperative complications.

**Methods:**

Medline, Cochrane Library, Scopus, Web of Science, and EMBASE were searched for comparative studies till 16.04.2024, resulting in 679 articles, of which eight studies met inclusion criteria. Data on patient demographics, surgical technique, and perioperative outcomes was assessed. Cochrane Risk of Bias 2.0 (RoB 2.0) Tool and Newcastle-Ottawa Scale (NOS) were used for risk of bias assessment.

**Results:**

Two randomized controlled trials, one prospective, and five retrospective cohort studies with 358 patients in IIVCC+PM and 397 patients in PM alone group were included. IIVCC+PM resulted in significantly greater CVP reduction, less intraoperative blood loss (MD (95% CI) = − 233.03 (− 360.48 to − 105.58), *P* < 0.001), and less intraoperative blood transfusion (OR (95% CI) = 0.38 (0.25 to 0.57), *P* < 0.001) compared to PM alone. The two groups had comparable total operative time, transection time and total intraoperative fluid infusion. Patients undergoing IIVCC+PM had significantly shorter length of stay (MD (95% CI) = − 0.63 days (− 1.21 to − 0.05 days), *P* = 0.03) and overall complication rates (OR (95% CI) = 0.63 (0.43–0.92), *P* = 0.02) compared to PM alone group.

**Conclusion:**

The utilization of IIVCC along with PM during liver resection may be beneficial in reducing intraoperative bleeding and blood transfusion without adversely influencing operative times or perioperative outcomes compared to PM alone.

**Supplementary Information:**

The online version contains supplementary material available at 10.1007/s00423-024-03344-6.

## Introduction

Hepatectomy is a common surgical procedure, which is often utilized as a curative measure for a range of benign as well as malignant liver pathologies. Despite improvements in imaging modalities, surgical techniques and perioperative management, intraoperative bleeding poses a significant challenge during hepatectomy. During major hepatic resection, intraoperative blood loss may range from 200 to 2000 mL, with approximately 25.2–56.8% of patients requiring intraoperative blood transfusion [1, 2]. Several studies have identified intraoperative blood loss and transfusions, during hepatectomy, as significant risk factors for perioperative morbidity and mortality [3]. Therefore, it remains crucial to identify and implement interventions to minimize intraoperative bleeding during hepatectomy.

Contemporary evidence suggests that maintaining CVP of ≤5cmH2O provides optimal conditions to minimize retrograde bleeding from hepatic veins during hepatectomy [4]. Currently, anaesthesiological interventions such as postural management, fluid restriction, diuretics and vasodilatory medications are commonly used to achieve optimal CVP [5]. Although such interventions are effective in reducing blood loss, they may, theoretically, result in hemodynamic instability and impair renal and cardiac function [6].

On the other hand, surgical techniques such as hepatic inflow control via hepatoduodenal ligament clamping, i.e., Pringle maneuver (PM) are commonly utilized with the aim of reducing intraoperative bleeding. However, recent evidence has argued against its utility, suggesting that it is not as beneficial in minimizing bleeding as previously thought [7, 8]. A potential explanation for these findings may be related to the retrograde bleeding from the patent hepatic veins and liver sinusoids, especially if CVP is not appropriately managed [9].

Recently, surgical techniques such as infrahepatic inferior vena cava clamping (IIVCC) have been increasingly utilized as a means of controlling CVP and reducing intraoperative bleeding. Rahbari et al. have shown that IIVCC may be more effective, in comparison to anaesthesiological interventions alone, in reducing intraoperative bleeding while minimizing the risk of hemodynamic instability [6]. Several studies have also assessed the feasibility of IIVCC combined with PM in reducing parenchymal bleeding during hepatic transection [10–13]. Conversely, some studies have also emphasized that IIVCC may also result in significant intraoperative hypotension and increase the risk of pulmonary embolism [6, 14].

The aim of the meta-analysis was to assess the perioperative outcomes of combined IIVCC+PM in comparison to PM alone approach in patients undergoing hepatic resection.

## Methods and materials

The meta-analysis was conducted and reported in accordance with PRISMA (Preferred Reporting Items for Systematic Reviews and Meta-Analyses) statement. The protocol for the study detailing the research question, search strategy, criteria for inclusion and risk of bias assessment was established a priori. The protocol was not registered with PROSPERO.

### Eligibility criteria

Observational studies and randomized controlled trials (RCT) were considered eligible for the review if published in English. Studies comparing IIVCC+PM approach with PM alone approach in patients undergoing laparoscopic or open hepatectomy were eligible for inclusion in the review. If PM was not performed for all patients and left at the surgeon’s discretion, the studies were excluded to allow appropriate comparison between IIVCC+PM and PM alone groups. Additionally, studies comparing IIVCC with anaesthesiological interventions for CVP reduction or no intervention controls groups were excluded. The primary outcomes of interest were related to transection time, total operation time, amount of intraoperative IV fluid infusion and need for intraoperative blood transfusion (IOBT). Secondarily, intraoperative outcomes including total blood loss (TBL) and blood loss (BL) during transection and postoperative outcomes such as length of hospital stay (LOS), overall and severe (Clavien-Dindo Grade ≥ 3) complications, and early mortality were also assessed. Additionally, the effect of IIVCC clamping on CVP was considered if reported in included studies.

### Search strategy and selection process

**Fig. 1:**
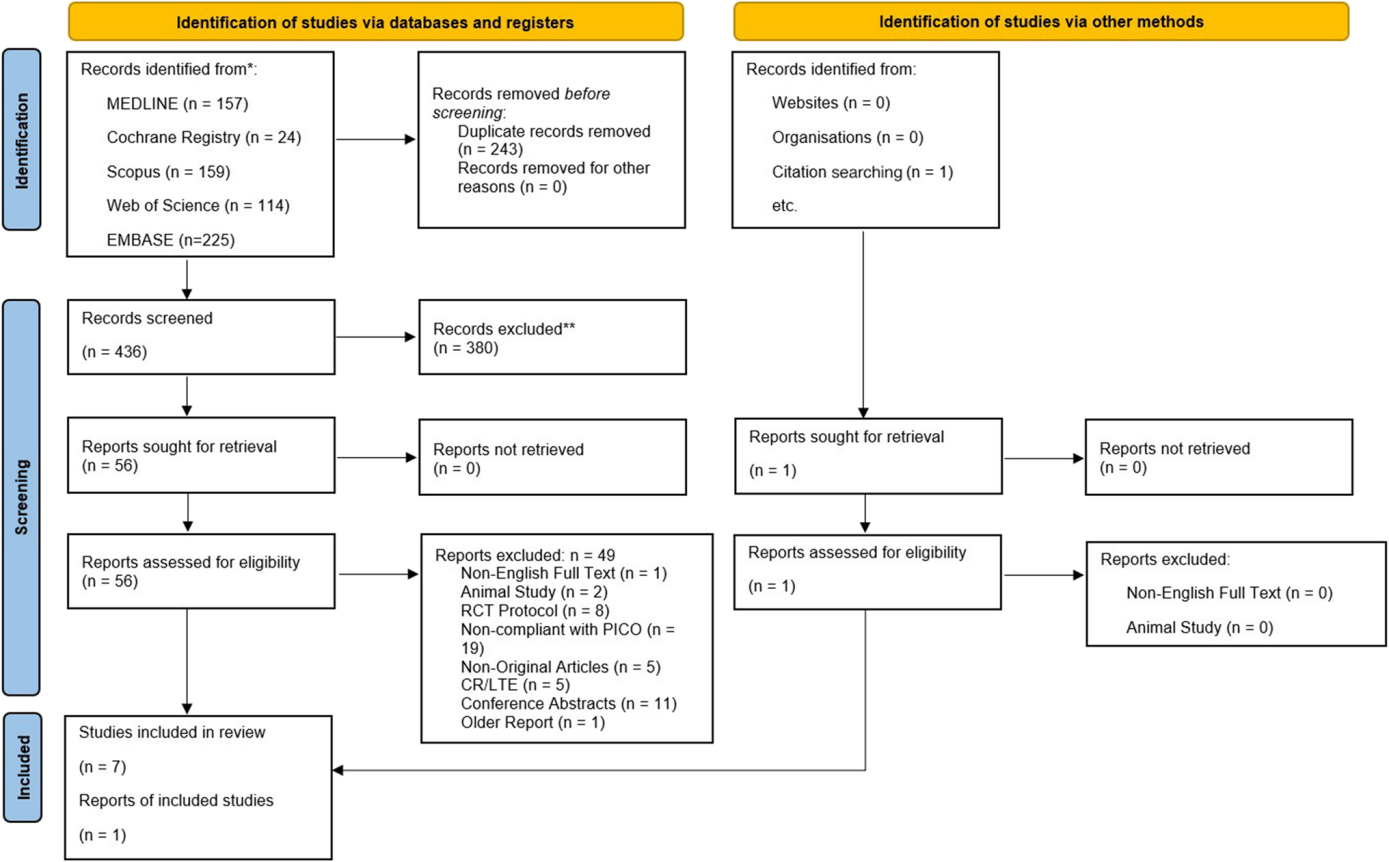
PRISMA Flowchart

A comprehensive literature search of Embase, MEDLINE, Web of Science, Scopus, and the Cochrane Central Register of Controlled Trials (CENTRAL) databases was performed from inception through to April 2024 to identify full-text articles related to the research question. The following search strategy, without any filters, was utilized: *(“infrahepatic inferior vena cava” OR “infra-hepatic inferior vena cava” OR “inferior vena cava” OR “IVC”) AND (“clamping”) AND (“hepatic resection” OR “liver resection” OR “hepatectomy” OR “hepatic dissection”)*. Backward citation chaining of relevant full-text articles was conducted to identify additional eligible full-text articles. The PRISMA flowchart describing the results of search and screening process is provided as Fig. 1.

After the initial search, identified articles were screened based on titles and abstract by two independent authors (AP, JT). Full text of eligible articles was reviewed against pre-specified inclusion criteria for the meta-analysis. Any disagreements between the reviewers were resolved through discussion with a third author and consensus.

### Data extraction

A structured data collection form was used by two independent reviewers (AP, JT) to extract data from included studies. Data regarding baseline study and patient characteristics, surgical and occlusion (IIVCC or PM) technique, intraoperative outcomes, changes in CVP, postoperative outcomes and funding sources were retrieved.

### Risk of Bias assessment

Cochrane Risk of Bias 2.0 (RoB 2.0) Tool and Newcastle-Ottawa Scale (NOS) were used to assess risk of bias within included RCT and observation studies, respectively [15, 16]. The risk of bias assessment was carried by two authors (AP, JT) independently with any disagreements resolved through consensus. The publication bias, using funnel plot, was not assessed as the meta-analysis did not include more than 10 studies.

### Statistical analysis

Pooled summary estimates for continuous variables were performed using mean difference (MD) and for categorical variables using odds ratio (OR) with respective 95% confidence interval (CI). If studies reported continuous variables as median (interquartile range), Wang et al’s method was utilized for conversion into estimated mean ± standard deviation and used in the statistical analysis [17]. Additionally, weighted arithmetic means (WAM) were calculated for selected categorical outcomes to establish pooled summary rates. Heterogeneity between studies was measured using the I^2^ statistic, with I^2^ > 50% considered to indicate significant statistically heterogeneity. Summary estimates were produced using random-effects model (in case of significant heterogeneity) or fixed-effects model. Forest plots were generated for visual representation of analyzed outcomes. Review Manager 5.0 (Cochrane Collaboration, London, United Kingdom) was used to conduct all statistical analysis.

## Results

The initial search identified 679 articles, of which, eight studies were included in the meta-analysis. Of these, five were retrospective cohort studies [12–14, 18, 19], one prospective cohort study [20], whereas two were randomized controlled trials [10, 11]. The summary of study features and baseline characteristics of the patient cohort is provided in Table 1. A total of 755 patients were included in the meta-analysis, with 358 patients in the IIVCC+PM group and 297 patients in the PM alone group.

**Table 1:** Baseline patient characteristics of included studies

Author	Study Design	Grouping	N patient	Male%	Age (Years)	Cirrhosis (N, %)	Tumour Size (cm)	Number of tumours (N)
Xiao et al. 2021, China	R	IIVCC+PM	68	54.4	56.8 ± 9.3	48 (71%)	4.3 ± 1.6	NR
		PM	64	53.1	57.2 ± 8.9	48 (75%)	4.5 ± 1.9	NR
Uchiyama et al. 2009, Japan	R	IIVCC+PM	20	75.0	61.6 ± 12.9	5 (25%)	NR	NR
		PM	58	75.9	64.8 ± 16.8	14 (24%)	NR	NR
Yang et al. 2013, China	R	IIVCC+PM	60	71.7	48.7 ± 10.8	30 (50%)	7.5 ± 3.4	Single/Multiple: 47/13
		PM	53	71.7	49.5 ± 12.1	28 (53%)	6.9 ± 2.3	Single/Multiple: 42/11
Zhang et al. 2017, China	R	IIVCC+PM	15	73.3	44.2 ± 8.5	NR	14.8 ± 6.2	Single/Multiple: 11/4
		PM	21	81.0	46.2 ± 10.3	NR	12.9 ± 3.4	Single/Multiple: 15/6
Otsubo et al.2004, Japan	R	IIVCC+PM	47	63.8	59.3 ± 14.4	NR	NR	NR
		PM	56	73.2	55.4 ± 16.1	NR	NR	NR
Ueno et al. 2016, Japan	RCT	IIVCC+PM	45	75.6	69.0 ± 10.7	11 (24%)	NR	NR
		PM	45	68.9	70.0 ± 9.9	14 (31%)	NR	NR
Kato et al. 2008, Japan	RCT	IIVCC+PM	43	NR	60.0 ± 12.4	NR	NR	Single/Multiple: 28/15
		PM	42	NR	62.8 ± 9.4	NR	NR	Single/Multiple: 27/15
Chen et al. 2006, China	P	IIVCC+PM	60	88.3	39.7 ± 3.6	60 (100%)	11.5 ± 1.9	Single/Multiple: 48/12
		PM	58	87.9	41.5 ± 4.1	58 (100%)	10.4 ± 2.6	Single/Multiple: 48/10

*R* retrospective, *RCT* randomised control trial, *P* prospective, *IIVCC* intrahepatic inferior vena cava clamping, *PM* Pringle maneuveur, *NR* not reported.

In the majority of studies, primary HCC was the most common indication for resection. Details relating to surgical technique are presented in Table 2. However, one study included patients undergoing resection for only benign indications [18]. Open hepatectomy was performed in six included studies while two performed laparoscopic resection. The weighted arithmetic means of patients undergoing major hepatectomy was 63% and 63.06% in IIVCC+PM compared to PM alone group, respectively. The PM time ranged from 10 to 20 minutes with 5 min break, across studies. In the five studies reporting average PM duration, it ranged from 12.6–58.3 minutes in the IIVCC+PM group and 13.5–65.6 minutes in the PM alone group [11–13, 18, 20]. Only four studies reported the average IIVCC duration with it ranging from 5.5–66.5 minutes [12, 13, 18, 20]. The criteria for inclusion and exclusion of patients within each study is presented in Table S1.

**Table 2:** Details of surgical intervention from the included studies

Author	Grouping	Indication (N)	Surgical Approach	Extent of Liver Resection (N)	Resection Equipment	Pringle Technique	PM Duration (mins)	IIVCC Technique	IIVCC Duration (mins)
Xiao et al. 2021, China	IIVCC+PM	Primary (52), Metastatic (7), Benign (9)	Laparoscopic	Minor (33), Major (35)	Harmonic Scalpel	15 mins with 5 mins break	NR	Partial	NR
	PM	Primary (48), Metastatic (5), Benign (11)		Minor (26), Major (38)			NR		NR
Uchiyama et al. 2009, Japan	IIVCC+PM	Primary (9), Metastatic (6), Cholangiocarcinoma (3), Benign (2)	Open	Minor (8), Major (12)	Ultrasonic Dissector	20 mins with 5 mins break	50.8 ± 17.8	Partial	66.5 ± 18.8
	PM	Primary (31), Metastatic (20), Cholangiocarcinoma (4), Benign (3)		Minor (16), Major (42)			52.6 ± 19.7		NR
Yang et al. 2013, China	IIVCC+PM	Primary (39), Metastatic (7), Cholangiocarcinoma (7), Benign (7)	Open	Minor (27), Major (29), Others (4)	Kelly Forceps (Clamp Crush Technique)	15 mins with 5 mins break	22.6 ± 7.1	Complete	28.2 ± 2.4
	PM	Primary (32), Metastatic (7), Cholangiocarcinoma (9), Benign (5)		Minor (19), Major (29), Others (5)			18.9 ± 5.8		NR
Zhang et al. 2017, China	IIVCC+PM	Benign (15)	Laparoscopic	NR	Harmonic Scalpel	NR	26.4 ± 5.7	Complete	14.2 ± 1.6
	PM	Benign (21)		NR			31.3 ± 6.2		0
Otsubo et al.2004, Japan	IIVCC+PM	Primary (28), Metastatic (10), Cholangiocarcinoma (5), Others (4)	Open	Major (47)	CUSA	10 mins with 5 mins break	NR	Partial	NR
	PM	Primary (31), Metastatic (14), Cholangiocarcinoma (4), Others (7)		Major (56)			NR		NR
Ueno et al. 2016, Japan	IIVCC+PM	Primary (28), Metastatic (7), Cholangiocarcinoma (8), Benign (1), Gallbladder (1)	Open	Minor (25), Major (20)	CUSA	20 mins with 5 mins break	NR	Partial	NR
	PM	Primary (29), Metastatic (8), Cholangiocarcinoma (6), Benign (2)		Minor (23), Major (22)			NR		NR
Kato et al. 2008, Japan	IIVCC+PM	Primary (35), Metastatic (6), Cholangiocarcinoma (1), Benign (1)	Open	Minor (24), Major (19) ^•^	CUSA	15 mins with 5 mins break	58.3 ± 21.3	Complete	NR
	PM	Primary (34), Metastatic (7), Gallbladder (1)		Minor (24), Major (18) ^•^			65.6 ± 34.5		NR
Chen et al. 2006, China	IIVCC+PM	Primary (60)	Open	Minor (7), Major (53)	Kelly Forceps (Clamp Crush Technique)	NR	12.6 ± 2.7	Complete	5.5 ± 2.2
	PM	Primary (58)		Minor (8), Major (49)			13.5 ± 2.1		0

*IIVCC* intrahepatic inferior vena cava clamping, *PM* Pringle maneuveur, *CUSA* Cavitron Ultrasonic Surgical Aspirator, *NR* not reported

### Risk of bias assessment

Based on the NOS, the quality of the six included cohort studies was deemed good, with NOS score ranging from 7 to 9. The overall risk of bias, based on RoB 2.0, was “some concerns” for the Kato et al. study due to lack of information on allocation concealment and lack of blinding, while it was deemed “low” for Ueno et al. [10, 11].

### Intraoperative outcomes

**Fig. 2:**
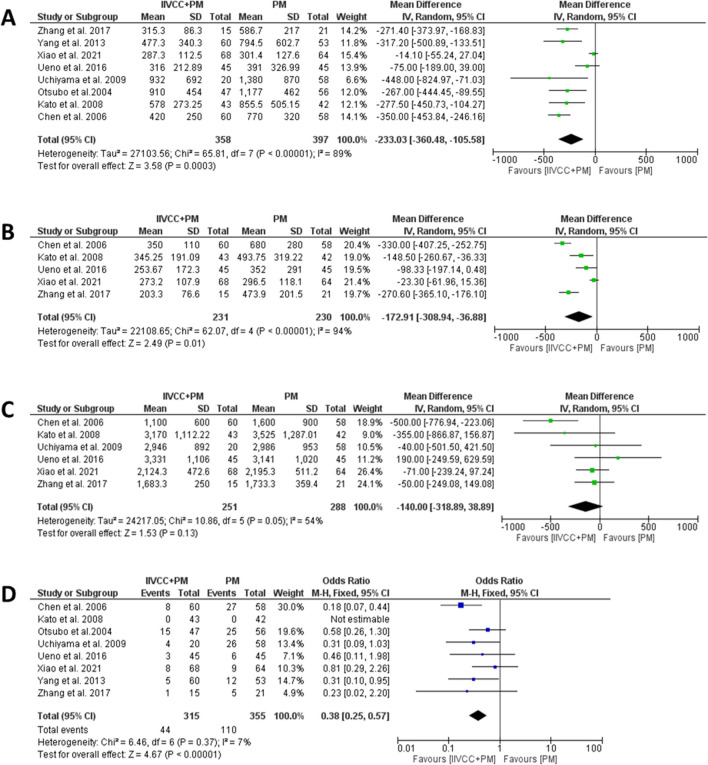
Forest plots comparing intraoperative outcomes between IIVCC+PM and PM – (**a**) Total blood loss, (**b**) Blood loss during transection, (**c**) Total intravenous fluid infusion, and (**d**) need for intraoperative blood transfusion

**Fig. 3:**
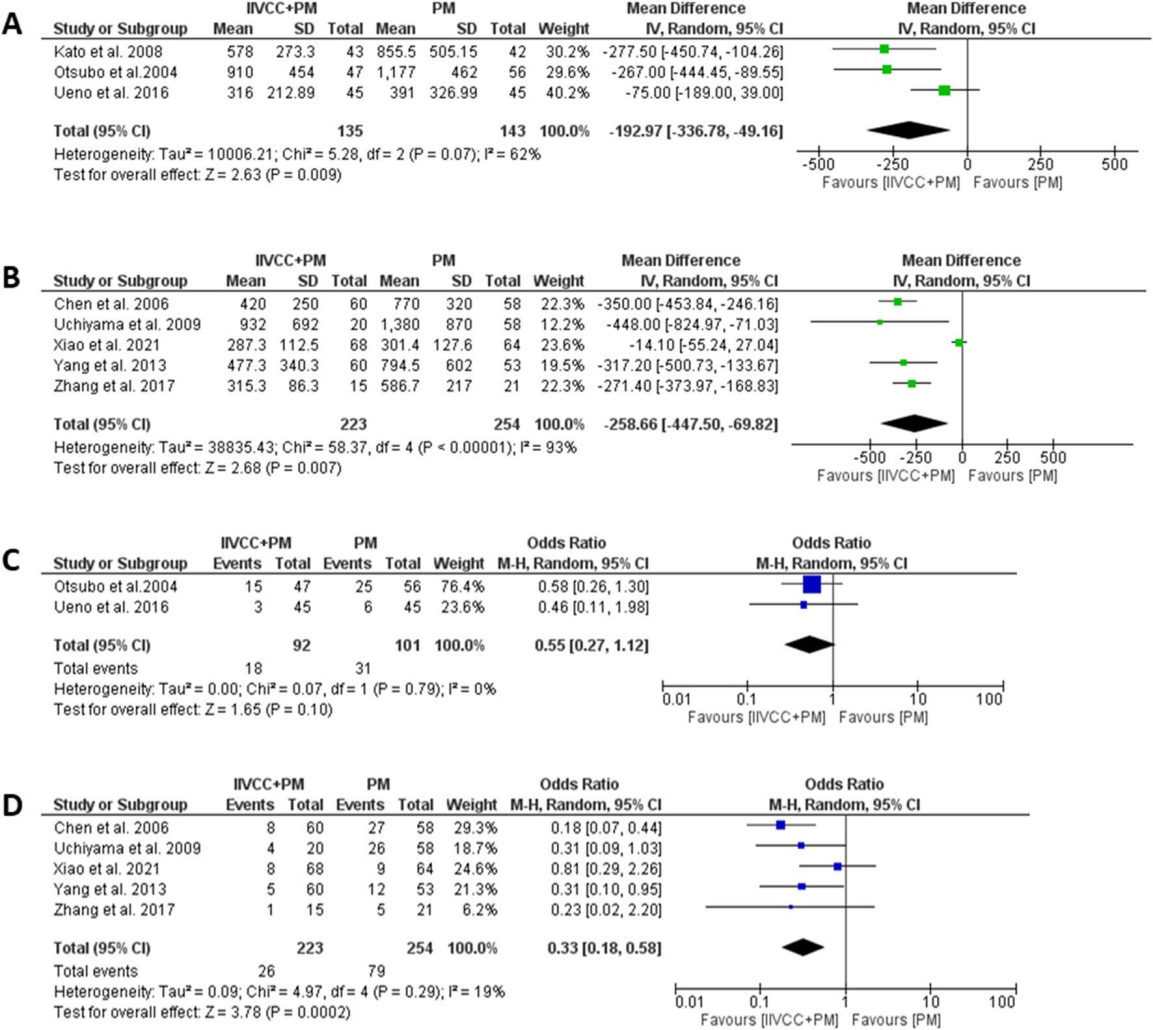
Forest plots on subgroup analysis of intraoperative outcomes based on transection technique – Total blood loss in (**a**) studies using Cavitron Ultrasonic Surgical Aspirator (CUSA) and (**b**) using harmonic scalpel or Kelly forceps, need for intraoperative blood transfusion (**c**) in studies using CUSA and (**d**) in studies using harmonic scalpel or Kelly forceps

The details relating to intraoperative outcomes are presented in Table 3. All studies provide information on TBL, however, only four studies explained the method of estimating TBL ((suction canister volume – irrigation fluids) + (soaked gauze weight – dry gauze weight)). Patients in the IIVCC+PM group experienced significantly less TBL in comparison to PM alone group (MD (95% CI): -233 mL (− 360 to -106 mL), I^2^ = 89%., *P* < 0.001). (Fig. 2a) Five studies reported on BL during transection, which was found to be significantly less in the IIVCC+PM group compared to PM alone group (MD (95% CI): -173 mL (− 309 to -37 mL), I^2^ = 94%. *P* = 0.01). (Fig. 2b) In a subgroup analysis of parenchymal transection with cavitron ultrasonic surgical aspirator (CUSA), IIVCC+PM group was found to experience significantly less total blood loss in comparison to PM alone group (MD (95%CI): − 192.97 mL (− 336.78 to − 49.16 mL), I^2^ = 62%, *P* = 0.009). (Fig. 3a) Similarly, analysis of studies using Harmonic scalpel and Kelly forceps also revealed significantly less TBL with IIVCC+PM compared to PM alone group (MD (95%CI): − 258.66 ml [− 447.50 to − 69.82 ml], I^2^ = 93%, *P* = 0.007). (Fig. 3b) However, the amount of intraoperative IV fluid transfusion was comparable between IIVCC+PM and PM alone groups (MD (95% CI): -140 mL (− 319 to 39 mL), I^2^ = 54%. *P* = 0.13). (Fig. 2c).

**Table 3:** Data on Intraoperative Outcomes from the included studies

Author	Grouping	N Patient	TBL (mL)	BL during Transection (mL)	Parenchymal Transection Duration (mins)	Intraoperative IV Fluid Infusion (mL)	Need for IOBT (N, %)	Total Operative Time (mins)
Xiao et al. 2021, China	IIVCC+PM	68	287.3 ± 112.5	273.2 ± 107.9	159.6 ± 71.8	2124.3 ± 472.6	8 (11.8%)	185.7 ± 61.2
	PM	64	301.4 ± 127.6	296.5 ± 118.1	165.3 ± 69.2	2195.3 ± 511.2	9 (14.1%)	196.3 ± 63.7
Uchiyama et al. 2009, Japan	IIVCC+PM	20	932.0 ± 692.0	NR	NR	2946.0 ± 892.0	4 (20.0%)	239.0 ± 43.0
	PM	58	1380.0 ± 870.0^*^	NR	NR	2986.0 ± 953.0	26 (45.0%)^*^	233.0 ± 58.0
Yang et al. 2013, China	IIVCC+PM	60	477.3 ± 340.3	NR	NR	NR	5 (8.3%)	178.1 ± 44.8
	PM	53	794.5 ± 602.7^*^	NR	NR	NR	12 (22.6%)^*^	166.6 ± 35.7
Zhang et al. 2017, China	IIVCC+PM	15	315.3 ± 86.3	203.3 ± 76.6	NR	1683.3 ± 250.0	1 (6.7%)	282.2 ± 53.0
	PM	21	586.7 ± 217.0^*^	473.9 ± 201.5^*^	NR	1733.3 ± 359.4	5 (23.8%)	259.5 ± 36.0
Otsubo et al.2004, Japan	IIVCC+PM	47	910.0 ± 454.0	NR	NR	NR	15 (31.9%)	NR
	PM	56	1177.0 ± 462.0^*^	NR	NR	NR	25 (41.6%)	NR
Ueno et al. 2016, Japan	IIVCC+PM	45	316.0 ± 212.9	253.7 ± 172.3	164.0 ± 67.0	3331.0 ± 1106.0	3 (6.7%)	365.0 ± 103.0
	PM	45	391.0 ± 326.9	352.0 ± 291.0^*^	191.0 ± 63.0	3141.0 ± 1020.0	6 (13.3%)	384.0 ± 116.0
Kato et al. 2008, Japan	IIVCC+PM	43	578.0 ± 273.3	345.3 ± 191.1	58.3 ± 21.3	3170.0 ± 1112.2	NR	NR
	PM	42	855.5 ± 505.2	493.8 ± 319.2	65.6 ± 34.5	3525.0 ± 1287.0	NR	NR
Chen et al. 2006, China	IIVCC+PM	60	420.0 ± 250.0	350.0 ± 110.0	13.6 ± 3.3	1100.0 ± 600.0	8 (13.3%)	133.0 ± 11.8
	PM	58	770.0 ± 320.0^*^	680.0 ± 280.0^*^	13.9 ± 3.8	1600.0 ± 900.0^*^	27 (46.5%)^*^	124.5 ± 10.7

*TBL* total blood loss, *BL* blood loss, *TSA* transection surface area, *BLV* blood loss volume, *TA* transection area, *IV* intravenous, *IOBT* intraoperative blood transfusion, *IIVCC* intrahepatic inferior vena cava clamping, *PM* Pringle maneuveur, *NR* not reported. ^*^P < 0.05, compared between two groups

Subsequently, the need of IOBT was found to be significantly less in IIVCC+PM than in PM alone group (OR (95% CI): 0.38 (0.25–0.57), I^2^: 7%, *P* < 0.001). (Fig. 2d) Moreover, subgroup analysis based on transection technique also found IIVCC+PM to significantly reduce the need for IOBT (CUSA: OR (95% CI): 0.55 (0.27 to 1.12), I^2^ = 0%, *P* = 0.10; Harmonic Scalpel/Kelly Forceps: OR (95% CI): 0.33 (0.18–0.58), I^2^ = 19%, *P* < 0.001). (Fig. 3c, d).

**Fig. 4:**
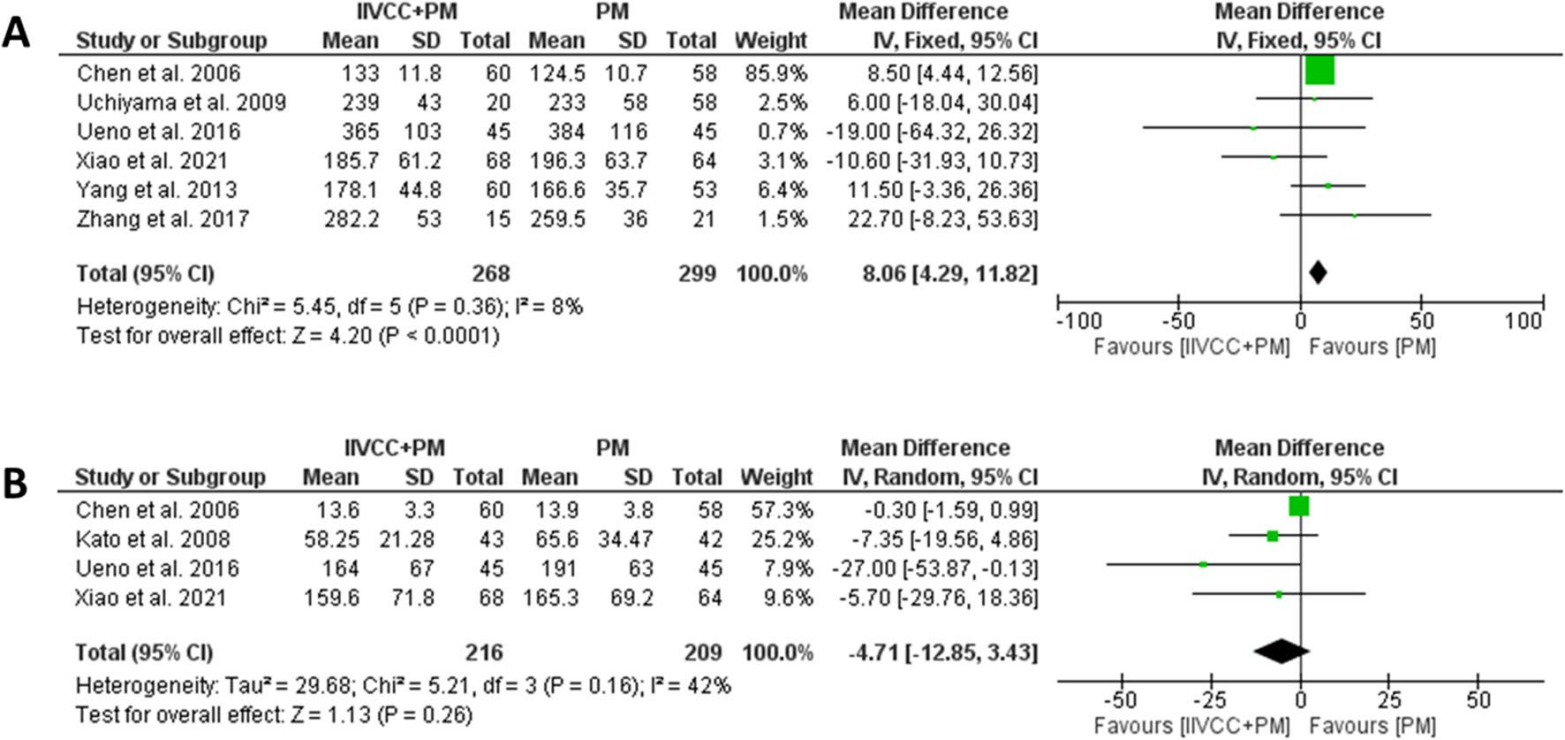
Forest plots comparing (**a**) total operative time and (**b**) parenchymal transection time between IIVCC+PM and PM groups

Only half of the articles reported on the parenchymal transection duration, which was found to be comparable between the groups (MD (95% CI): − 0.97 minutes (− 4.29 to 2.35 minutes), I^2^ = 79%, *P* = 0.57). (Fig. 4a) The total operative time was significantly shorter in patients undergoing IIVCC+PM than PM alone approach, however, the mean difference between the groups was approximately 8 minutes (MD (95% CI): − 8.1 minutes (4.3–11.8 minutes), I^2^: 8%, *P* < 0.001). (Fig. 4b).

Of the five articles reporting on CVP, pre-clamp CVP was similar between the IIVCC+PM and PM alone groups (MD (95% CI): 0.15 cmH2O (− 0.24 to 0.55 cmH2O), I^2^ = 0%, *P* = 0.45) [10, 11, 14, 19]. (Supplementary Fig. 1a) However, in-clamp CVP was significantly reduced with IIVCC+PM compared to PM alone (MD (95% CI): − 2.01 cmH2O (− 3.31 to − 0.72 cmH2O), I^2^ = 90%, *P* = 0.002). (Supplementary Fig. 1b) Only four studies reported information on post-clamp CVP, with two studies demonstrating similar post-clamp CVP between the groups [19, 20]. (Supplementary Table 2).

### Postoperative outcomes

**Fig. 5:**
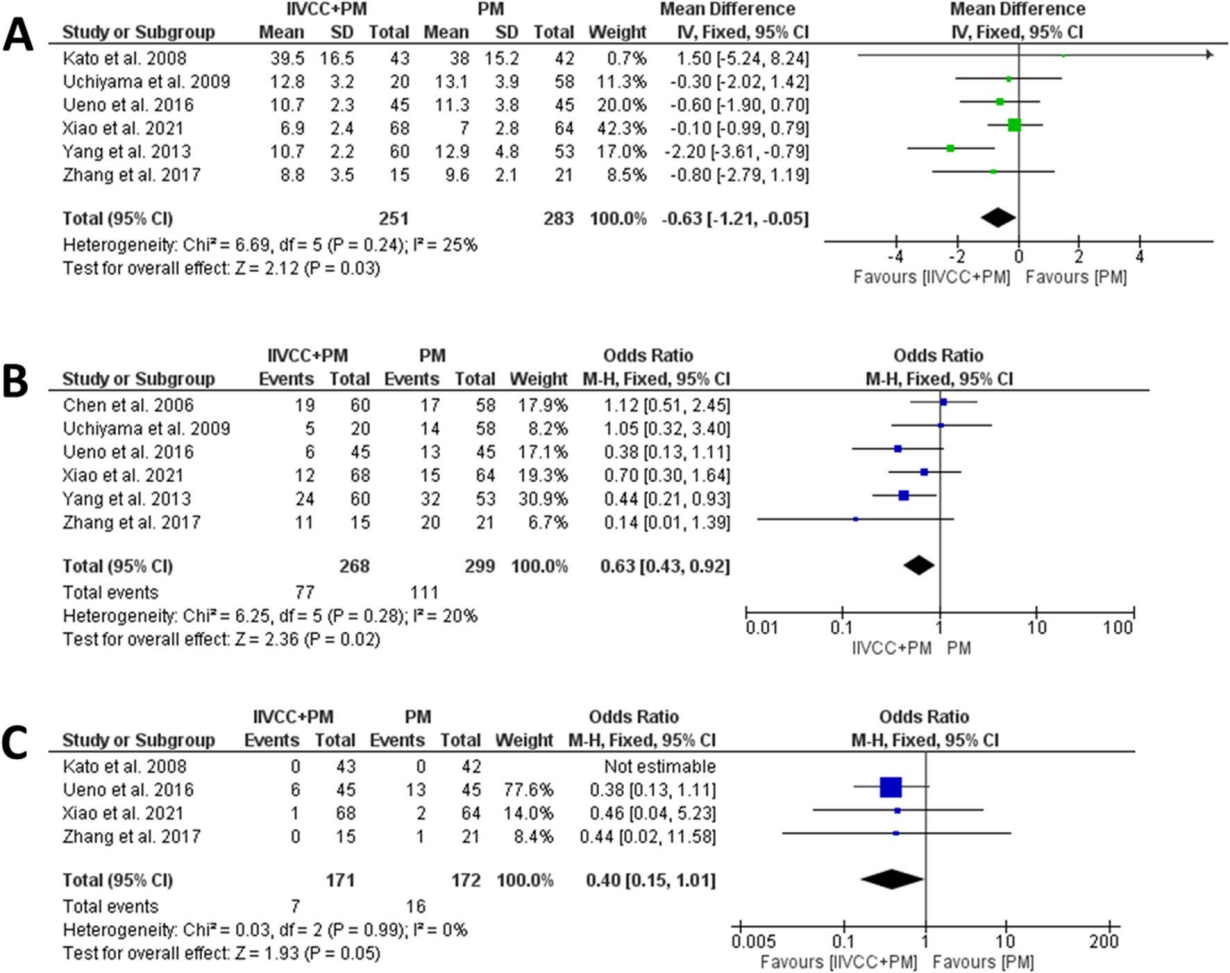
Forest plots on postoperative outcomes (**a**) length of hospital stay, (**b**) overall morbidity, and (**c**) major (Clavien-Dindo Grade ≥ 3) morbidity

The summary of postoperative outcomes is detailed in Table 4. Patients undergoing hepatectomy with IIVCC+PM had significantly lower LOS compared to the PM alone cohort (MD (95% CI): 0.63 days (− 1.21 to − 0.05 days), I^2^: 25%, *P* = 0.03). (Fig. 5a) Additionally, patients in the IIVCC+PM group experienced significantly lower overall complications than those in the PM alone group (OR (95% CI): 0.63 (0.43–0.92), I^2^: 20%, *P* = 0.02). (Fig. 5b) The major complication rates were also comparable between the two groups (OR (95% CI): 0.40 (0.15–1.01), I^2^: 0%, *P* = 0.05). (Fig. 4c) Only one case of mortality was reported in a PM cohort due to hepatic insufficiency [20].

**Table 4:** Data on Postoperative Outcomes from the included studies

Author	Grouping	N Patient	Hospital Stay (days)	Overall Morbidity (N, %)	Major Complication Rates (CD ≥ Grade 3) (N)	Mortality (N, %)
Xiao et al. 2021, China	IIVCC+PM	68	6.9 ± 2.4	12 (17.6%)	1	0
	PM	64	7.0 ± 2.8	15 (23.4%)	2	0
Uchiyama et al. 2009, Japan	IIVCC+PM	20	12.8 ± 3.2	5 (25%)	NR	0
	PM	58	13.1 ± 3.9	14 (24.1%)	NR	0
Yang et al. 2013, China	IIVCC+PM	60	10.7 ± 2.2	24 (40%)	NR	0
	PM	53	12.9 ± 4.8^*^	32 (60.4%)^*^	NR	0
Zhang et al. 2017, China	IIVCC+PM	15	8.8 ± 3.5	11 (73.3%)	0	0
	PM	21	9.6 ± 2.1	20 (95.2%)	1	0
Otsubo et al.2004, Japan	IIVCC+PM	47	NR	NR	NR	0
	PM	56	NR	NR	NR	0
Ueno et al. 2016, Japan	IIVCC+PM	45	10.7 ± 2.3	6 (13.3%)	6	0
	PM	45	11.3 ± 3.8	13 (28.9%)	13	0
Kato et al. 2008, Japan	IIVCC+PM	43	39.5 ± 16.5	0	0	0
	PM	42	38 ± 15.2	0	0	0
Chen et al. 2006, China	IIVCC+PM	60	NR	19 (31.6%)	NR	0
	PM	58	NR	17 (29.3%)	NR	1 (1.72%)

*CD* Clavien-Dindo grade, *IIVCC* intrahepatic inferior vena cava clamping, *PM* Pringle manoeuvre, *NR* not reported. Major complications are defined as Clavien-Dindo Grade ≥ 3. ^*^P < 0.05, compared between two groups

## Discussion

Intraoperative bleeding during hepatectomy, and subsequently need for blood transfusion are important predictors of adverse perioperative outcomes [3, 21]. This meta-analysis identified that combination of IIVCC and PM is an effective surgical strategy to limit intraoperative blood loss and need for IOBT in comparison to PM alone approach. Additionally, our analysis demonstrated the safety of the combined approach, based on comparable operative and transection times as well as postoperative morbidity and mortality between IIVCC+PM and PM-alone group. These findings are in keeping with a previous meta-analysis on the topic [22]. However, it strengthens the evidence supporting the efficacy of IIVCC through a more stringent inclusion criteria (all patients undergoing PM with those in the study arm undergoing IIVCC additionally).

The improved efficacy of IIVCC+PM in reducing TBL and IOBT compared to PM alone can be attributed to the significant reduction in CVP achieved via IIVCC. In all included studies, pre-clamp CVP was comparable between the two groups. However, the majority of the included studies demonstrated a significant reduction from pre-clamp to in-clamp CVP with IIVCC+PM than PM-alone. Moreover, two studies found that the reduction in CVP during the “in-clamp” phase was significantly greater with IIVCC than PM alone [10, 23]. .In addition to reducing blood loss, low CVP also prevents vena cava distension and facilitates mobilization of liver and retrohepatic venous resection [4, 24]. Nonetheless, it is also important to note that with low CVP, there may be a considerable risk of intraoperative hemodynamic instability. In the cohort of Zhang et al., the mean arterial pressure (MAP) (93.5 ± 5.7 to 70.9 ± 6.1 mmHg, *P* < 0.001) significantly decreased, while HR (73.3 ± to 92.6 ± 11.0, *P* < 0.001) significantly increased after IIVCC+PM in comparison to pre-clamping levels [18]. Moreover, IIVCC may lead to poor perfusion of the hepatic parenchyma and impair renal function due to venous congestion (IVC is usually clamped proximal to renal veins) [18]. In our analysis, IIVCC was well tolerated, with only slight derangement of liver and renal function, which recovered in the early postoperative period without additional interventions.

Furthermore, our analysis showed that combined IIVCC+PM resulted in similar LOS, postoperative morbidity, and mortality in comparison to PM alone. Within the eight included studies, only one death was reported in PM alone group due to hepatic insufficiency [20]. Some of the most common complications reported included pleural effusion, ascites and bile leakage, with similar incidence between the two groups. Recently, there has also been a debate pertaining to the risk of developing venous thrombosis and pulmonary embolism (PE) with IIVCC [6, 25, 26], potentially due to the resulting hemostasis or dangerously low CVP. However, there were no cases of PE reported in any of the studies, suggesting the risks might not be significant. Previous studies have recommended the use of Trendelenburg’s position to prevent low CVP-induced embolic events, and suggested to avoid IIVCC in patients with CVP <5cmH2O to avoid air embolism [14, 27]. Additionally, Xiao et al., examining partial IIVCC, utilized intermittent pneumatic limb compression, and intraoperative ultrasonography to ensure IVC flow and detect thrombosis prior to release of IIVCC [19].

In addition to assessing the safety and feasibility of IIVCC, it is also important to consider its effect on various patient cohorts undergoing hepatectomy. Firstly, the risk of bleeding from hepatic veins may be greater during anatomical complex resections (except left lateral sectionectomy) as hepatic veins are often exposed on the cut surface during these procedures. The most important cause of excessive bleeding during liver resection is usually due to hepatic vein injury, which tends to be more difficult to control during laparoscopic resection. Ueno et al. demonstrated the feasibility of partial IIVCC in controlling such bleeding suggesting it as an ideal approach to utilize especially in resection of centrally located tumors neighboring/involving the main hepatic veins [10]. Secondly, it is important to consider the impact of IIVCC in patients with cirrhotic livers. It is widely accepted that cirrhotic livers are at significantly greater risk of intraoperative bleeding [28]. Chen et al., assessing only cirrhotic patients, demonstrated that IIVCC can be useful in controlling blood loss during hepatectomy and is well tolerated in patients with diseased liver [20]. Similarly, Ueno et al. and Xiao et al., in their subgroup analysis, found that IIVCC+PM was more beneficial in lowering CVP and decreasing intraoperative bleeding in patients with fibrotic or cirrhotic livers than those without a background of chronic liver disease [10, 19]. Therefore, IIVCC along with PM may be considered in patients undergoing complex anatomical hepatectomy and those with cirrhotic liver.

Additionally, it was also crucial to examine the technical aspects of IIVCC. The majority of studies included in this report performed open hepatectomy and found IIVCC+PM to be superior to PM alone approach. Two studies, Xiao et al. and Zhang et al., performed IIVCC during laparoscopic hepatectomy provided contradictory evidence. Zhang et al. showed that IIVCC+PM was effective in diminishing intraoperative bleeding, allowing for less extensive enucleation of hemangiomas, but resulting in greater hemodynamic compromise intraoperatively [18]. On the other hand, Xiao et al. examining hepatectomies for mainly liver malignancies found no difference in TBL between IIVCC+PM and PM alone group [19, 29]. Although these studies concluded that laparoscopic IIVCC is safe and feasible, further studies with homogenous indication and surgical technique are needed. It is also important to consider the degree of IIVCC (complete versus partial) undertaken. In this report, four studies performed partial IIVCC while the remaining performed complete clamping of the IVC. Complete IIVCC carries significant hazard of major central hypovolemia with resultant hepatic, cardiac and renal insult. Xiao et al. highlighted that the main benefit of partial IIVCC is the adjustability of the clamping duration and strength guided by hemodynamic parameters. Additionally, in case of severe hemorrhage requiring intensive fluid infusion to maintain hemodynamic stability, partial IIVCC may be better able to reduce CVP and enable hemostatic control [23].

Moreover, it is vital to ascertain the duration and method (intermittent versus continuous) of IIVCC in combination with PM which can be safely performed. Effective clamping techniques are essential in sufficiently reducing intraoperative bleeding. Chen et al. performed the shortest mean duration of IIVCC (5.5 ± 2.2 mins) and PM (12.6 ± 2.7 to 13.5 ± 2.1 mins), with expectedly greatest amount of blood loss during transection in both groups relative to other studies [20]. Previous studies have shown efficacy of continuous PM (cumulative clamping time 30–50 min) in improving intraoperative and postoperative outcomes [30]. None of the studies in this series identified a suitable cut off for IIVCC, but Uchiyama et al. provide a proof of concept that prolonged, continuous IIVCC (mean duration = 66.5 ± 18.8 mins) may be feasible and likely allow effectively bloodless resection [12]. Additionally, all the studies utilized an intermittent PM technique, combining IIVCC with continuous PM may offer further improvement in hemostatic control, but with potentially increased risk of ischemic hepatic injury. Therefore, it is necessary through further research to identify the ideal clamping time and method, which balances the risk of bleeding versus hemodynamic compromise and morbidity [30, 31]. Furthermore, in our subgroup analyses based on resection equipment (CUSA and harmonic scalpel + crush clamping), IIVCC+PM approach was associated with significant reduction in TBL and IOBT regardless of the transection equipment used. However, the study samples in each subgroup analysis were limited, therefore, future studies should consider the effect of transection devices in combination with vascular clamping techniques.

This meta-analysis describes the largest evidence base on IIVCC+PM in comparison to PM alone approach. However, it is important to consider the following limitations. Firstly, the studies were heterogenous in terms of vascular occlusion (technique and duration) and parenchymal transection techniques. Based on the reporting surgical technique, it was difficult to ascertain if complete or partial, and if continuous or intermittent IIVCC clamping was performed in some studies. Therefore, it was difficult to determine the influence of various IIVCC techniques on surgical outcomes. Secondly, patients with severe comorbidities (heart disease, renal dysfunction, venous thrombosis) were excluded. Therefore, it is possible that better outcomes with IIVCC may be related in part due to selection bias. Additionally, the included studies examined patients with various indications including primary/metastatic malignancy and benign conditions. The indications for hepatectomy may also affect the assessed outcomes such as morbidity and mortality. However, a subgroup analysis to assess the efficacy of IIVCC+PM of each indication was not feasible due to a lack of data on individual indications. Lastly, none of the studies reported on long-term benefits of the combined approach.

In conclusion, IIVCC appears to be a safe and effective technique to reduce CVP, and thereby the TBL and subsequent need for IOBT, without substantial increase in operative and transection time as well as perioperative complications in patients undergoing hepatectomy. This benefit of IIVCC remains regardless of the hepatic parenchyma transection technique utilized.

### Supplementary information

ESM 1 Supplementary Fig. 1: Forest plots comparing (a) pre-clamp central venous pressure and (b) in-clamp central venous pressure between IIVCC+PM and PM groups (TIF 311 kb)

ESM 2 (DOCX 20 kb)

ESM 3 (PDF 65 kb)
