# Supplementary figures and images for: Perioperative outcomes of utilizing infrahepatic inferior vena cava clamping and Pringle maneuver during hepatectomy: a meta-analysis
Source: Langenbecks Arch Surg. 2024 May 17;409(1):160. doi: 10.1007/s00423-024-03344-6 (PMC11101571; doi:10.1007/s00423-024-03344-6)

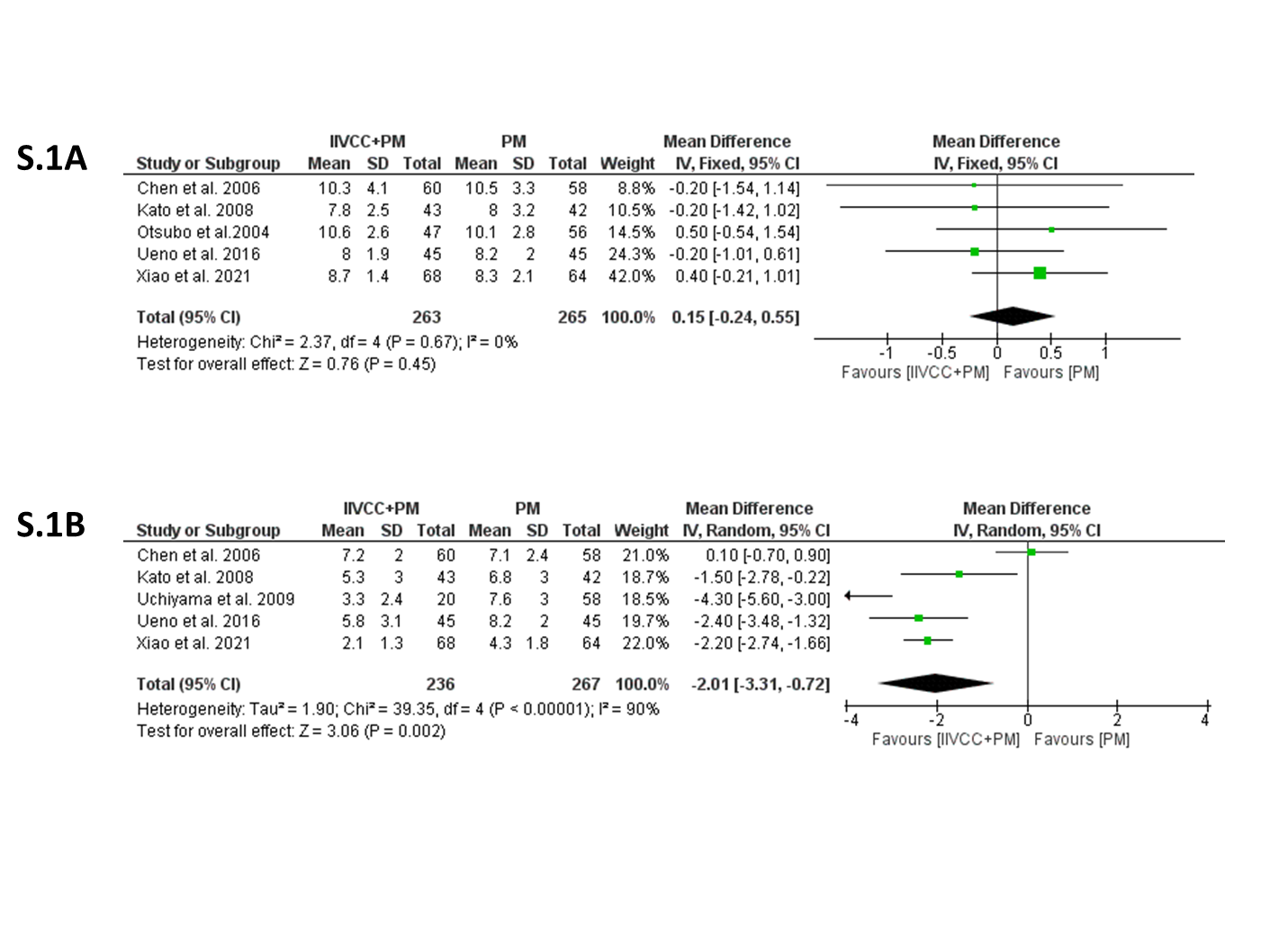

Supplement: Supplementary file 1 — Supplementary Fig. 1: Forest plots comparing (a) pre-clamp central venous pressure and (b) in-clamp central venous pressure between IIVCC+PM and PM groups (TIF 311 kb) [file 423_2024_3344_MOESM1_ESM.tif]
