# Supplementary material for: Perioperative outcomes of utilizing infrahepatic inferior vena cava clamping and Pringle maneuver during hepatectomy: a meta-analysis
Source: Langenbecks Arch Surg. 2024 May 17;409(1):160. doi: 10.1007/s00423-024-03344-6 (PMC11101571; doi:10.1007/s00423-024-03344-6)
Supplement: Supplementary file 2 — (DOCX 20 kb) [file 423_2024_3344_MOESM2_ESM.docx]

**Title:** Intraoperative Outcomes using Infrahepatic Inferior Vena Cava Clamping during Hepatectomy: A Meta-analysis

Jacob Tan^1*^, Agastya Patel^1,2*^, Joel Lambert^1^, Samuel Kitching^1^, Affan Iqbal^1^, Thomas Satyadas^1^

1. Regional Hepato-Pancreato-Biliary Surgical Unit, Manchester Royal Infirmary, Manchester, United Kingdom
2. Department of General, Endocrine and Transplant Surgery, Medical University of Gdansk, Gdansk, Poland

^*^Equal contributors

**Corresponding Author**

Agastya Patel, M.D.

Regional Hepato-Pancreato-Biliary Surgical Unit,

Manchester Royal Inﬁrmary, Manchester M13 9WL, United Kingdom

Email: agastya.patel@mft.nhs.uk

**Supplementary table 1: Inclusion/Exclusion criteria**

| Author | Inclusion/Exclusion Criteria |
| --- | --- |
| Xiao et al. 2021, China | Inclusion: Tumour location (confined to liver segment, lobe or hemiliver without major vascular invasion), FLR 40%, Child–Pugh A, ICG-R15 < 20%, ASA I to III  Exclusion: Extrahepatic metastases, Hepatic vascular invasion, Hypersplenism, Severe oesophageal/gastric varices, Atrial fibrillation, Renal insufficiency |
| Uchiyama et al. 2009, Japan | Exclusion: Concomitant bowel resection, Contralateral hepatic resection |
| Yang et al. 2013, China | Inclusion: Tumours involving hepatocaval confluence, Child-Pugh A for major hepatectomy  Exclusion: Extrahepatic metastases, Peripheral lesion not involving hepatocaval confluence, Caudate lobe lesion, Severe comorbidities |
| Zhang et al. 2017, China | Inclusion: Haemangiomas adjacent to the main portal pedicle, major hepatic veins or IVC |
| Otsubo et al.2004, Japan | Inclusion: CVP >5cmH2O, Right/left hemihepatectomy between 1995-2000, Consent given after thorough explanation of operative procedure |
| Ueno et al. 2016, Japan | Inclusion: 20-80 years old for anatomic liver resection, ECOG performance status 0–2, Child-Pugh A, Well-maintained full blood count and renal functions, Written informed consent, CVP ≥5 mm Hg before hepatectomy  Exclusion: CVP <5mmHg, Insufficient liver function, Previous laparotomy, NYHA III or higher, DVT, Severe COPD, RRT, Atrial fibrillation, Tricuspid regurgitation, Need for vascular reconstruction |
| Kato et al. 2008, Japan | Stratified by: Age (≥60 or <60), ICG-R15 (≥20% or <20%), Operative procedure (>1 or <1 Couinaud's segment), Number of tumours (single or multiple) |
| Chen et al. 2006, China | Inclusion: Large tumour (>5cm in diameter), Central lesion, No direct invasion of the hepatic hilar plate, Absent ascites or controllable with diuretics, Serum total bilirubin (TB) <1.2mg/dl Criteria for minor resection (<3segments): TB 0.7-1.2mg/dl, Albumin ≤3.5g/l, Cholesterol ≤3.6mmol/l, PT 3-4sec over control Criteria for major resection (≥3segments): TB ≤0.7mg/dl, Albumin>3.5g/l, Cholesterol >3.6mmol/l, PT <3sec over control, FLR 60%  Exclusion: Extrahepatic spread, requiring concomitant non-shunt operation, splenectomy, multiple liver resection, and extended right/left hepatectomy |

**Supplementary table 2: Outcomes relating to central venous pressure changes in relation to IIVCC+PM and PM.**

CVP, central venous pressure; pre-clamp: CVP before clamping; in-clamp, CVP during clamping period; post-clamp, CVP after declamping; IIVCC, intrahepatic inferior vena cava clamping; PM, Pringle maneuveur; NR, not reported.

* values reported as mean (interquartile range) in study. These are converted to mean±median.

• Values reported as mean (minimum-maximum) in this study. These are converted to mean±median.

~ P<0.05, compared between two groups.

| Author | Grouping | N patient | Pre-Clamp | In-Clamp | Post-Clamp | Difference (In-clamp - Pre-clamp) |
| --- | --- | --- | --- | --- | --- | --- |
| Xiao et al. 2021, China | IIVCC+PM | 68 | 8.7±1.4 | 2.1±1.3 | 7.8±1.9 | NR |
|  | PM | 64 | 8.3±2.1 | 4.3±1.8 ^~^ | 8.1±1.4 | NR |
| Uchiyama et al. 2009, Japan | IIVCC+PM | 20 | 7.5±2.4 | 3.3±2.4 | 7.1±2.3 | NR |
|  | PM | 58 | NR | 7.6±3.0 ^~^ | NR | NR |
| Yang et al. 2013, China | IIVCC+PM | 60 | 7.6±3.2 | 4.4±2.7 | NR | NR |
|  | PM | 53 | NR | NR | NR | NR |
| Zhang et al. 2017, China | IIVCC+PM | 15 | NR | NR | NR | NR |
|  | PM | 21 | NR | NR | NR | NR |
| Otsubo et al.2004, Japan | IIVCC+PM | 47 | 10.6±2.6 | 6.9±2.4 | NR | NR |
|  | PM | 56 | 10.1±2.8 | NR | NR | NR |
| Ueno et al. 2016, Japan | IIVCC+PM | 45 | 8.0±1.9 ^*^ | 5.8±3.1 ^*^ | 9.1±3.1 ^*^ | NR |
|  | PM | 45 | 8.2±2.0 ^*^ | 8.2±2.0 ^*, ~^ | NR | NR |
| Kato et al. 2008, Japan | IIVCC+PM | 43 | 7.8±2.5 ^•^ | 5.3±3.0 ^•^ | NR | -3.5±1.4 ^•, ~^ |
|  | PM | 42 | 8±3.2 ^•^ | 6.8±3.0 ^•^ | NR | -1.3±1.6 ^•^ |
| Chen et al. 2006, China | IIVCC+PM | 60 | 10.3±4.1 | 7.2±2.0 | 9.7±4.4 | NR |
|  | PM | 58 | 10.5±3.3 | 7.1±2.4 | 9.5±4.0 | NR |
